# The effect of plant domestication on host control of the microbiota

**DOI:** 10.1038/s42003-021-02467-6

**Published:** 2021-08-05

**Authors:** Riccardo Soldan, Marco Fusi, Massimiliano Cardinale, Daniele Daffonchio, Gail M. Preston

**Affiliations:** 1 University of Oxford, Department of Plant Sciences, Oxford, UK; 2 Edinburgh Napier University, School of Applied Sciences, Edinburgh, UK; 3 University of Salento, Department of Biological and Environmental Sciences and Technologies, Lecce, Italy; 4 King Abdullah University of Science and Technology (KAUST), Red Sea Research Center (RSRC), Thuwal, Saudi Arabia

**Keywords:** Microbiome, Evolutionary theory

## Abstract

Macroorganisms are colonized by microbial communities that exert important biological and ecological functions, the composition of which is subject to host control and has therefore been described as “an ecosystem on a leash”. However, domesticated organisms such as crop plants are subject to both artificial selection and natural selection exerted by the agricultural ecosystem. Here, we propose a framework for understanding how host control of the microbiota is influenced by domestication, in which a double leash acts from domesticator to host and host to microbes. We discuss how this framework applies to a plant compartment that has demonstrated remarkable phenotypic changes during domestication: the seed.

## Abstract (web summary)

Soldan and co-authors propose an evolutionary framework for understanding how host control of the microbiota is influenced by artificial selection. They go on to discuss the potential effect of domestication syndrome on the seed microbiome and plant-microbe interactions in the spermosphere.

## Introduction

According to FAO estimates, consumption of seeds comprises from 40 to 70% of both calories and protein in the human diet^1^, while most of the remaining 60 to 30% is constituted by derivates of animals, which again are mainly fed on seeds^2^. Each year, we produce about 2.5 billion tons of cereal grains (https://data.worldbank.org/indicator/ag.prd.crel.mt). This accomplishment can be considered to be the consequence of plant domestication^3,4^. In the last 12,000 years, humans have domesticated and semi-domesticated about 2500 plant species as crops^5,6^, which together with changes in agricultural practices, have enabled high yielding harvests. Many domesticated crops show common traits that distinguish them from their wild counterparts. This pool of common traits is called the domestication syndrome^7^ and includes increased fruit size and changes to reproductive strategy, branching, secondary metabolites, and seed-shattering^8,9^. The most remarkable domestication traits frequently involve the seed. For example, grain crops produce seeds that are up to 15.2 times bigger than seeds produced by their wild progenitors^10^.

Since the pioneering work of Darwin^11^ and lately Vavilov^12^ and Engelbrecht^13^, we have furthered our understanding of the origin of domestication^3,5,14^ and its genetic basis^9,15^. However, except for a few notable exceptions, little is still known about how plant domestication influences the community phenotypes of interacting organisms^16^. Among the different organisms that live in associations with plants, microorganisms have particular importance because of their contribution to plant growth and fitness^17–20^. These microbial communities are so closely associated with the plant that they can form, together with the plant host, a holobiont^21,22^. These intimate relationships are crucial for plant well-being, resistance, and resilience, as well as for basic processes of the plant life cycle, such as germination^23^. Since plant domestication, led by artificial selection, has shaped and changed many aspects of plant growth and development, it is logical to ask how these phenotypic changes have influenced the evolution of plant–microbe interactions. In recent years, we have witnessed an increasing research effort to understand the effect of plant domestication on the plant microbiome^24–28^ but evolutionary theories that could explain how the assembly of domesticated plant microbiomes differs from that of wild progenitors are still lacking.

In this work, we first adopt and expand the concept of the microbiome as an ecosystem on a “leash” using the framework proposed by Foster et al.^29^ to incorporate the effect of domestication, thus artificial selection and natural selection exerted by the agricultural ecosystem, on the host control of the microbiota. We then apply our framework for a specific plant compartment, the seed, and discuss the potential effect of plant domestication (phenotypic changes) on the seed microbiome and plant–microbe interactions in the spermosphere. We conclude by highlighting the implications of the double-leash framework from a biotechnological perspective.

## Domesticated plant microbiomes as an ecosystem on a double leash

To better explain the evolution of hosts and their microbiomes, Foster et al.^29^ propose that the microbiome can be broken down into three classes of effect (host-to-microbe, microbe-to-host, and microbe-to-microbe), each one with its evolutionary features. Because the host is under strong selective pressure to favour beneficial interactions with its microbiome^30^, and the host can control the environment in which its microbiome lives, the microbiome becomes “*a dynamic microbial ecosystem held on an ever-evolving leash by the host”*^29^. In this work, we adopt this framework with a particular focus on the host-to-microbe effects caused by domestication, thereby extending this concept to incorporate artificial selection (or direct selection) and natural selection (or indirect selection) acting through the agricultural ecosystem. Understanding the consequences of domesticated plant phenotypes on microbe-to-host effects requires an understanding of how these phenotypes could change host-to-microbe effects, particularly when domesticated plant phenotypes are often similar between different phylogenetically distant crops.

**Fig. 1:**
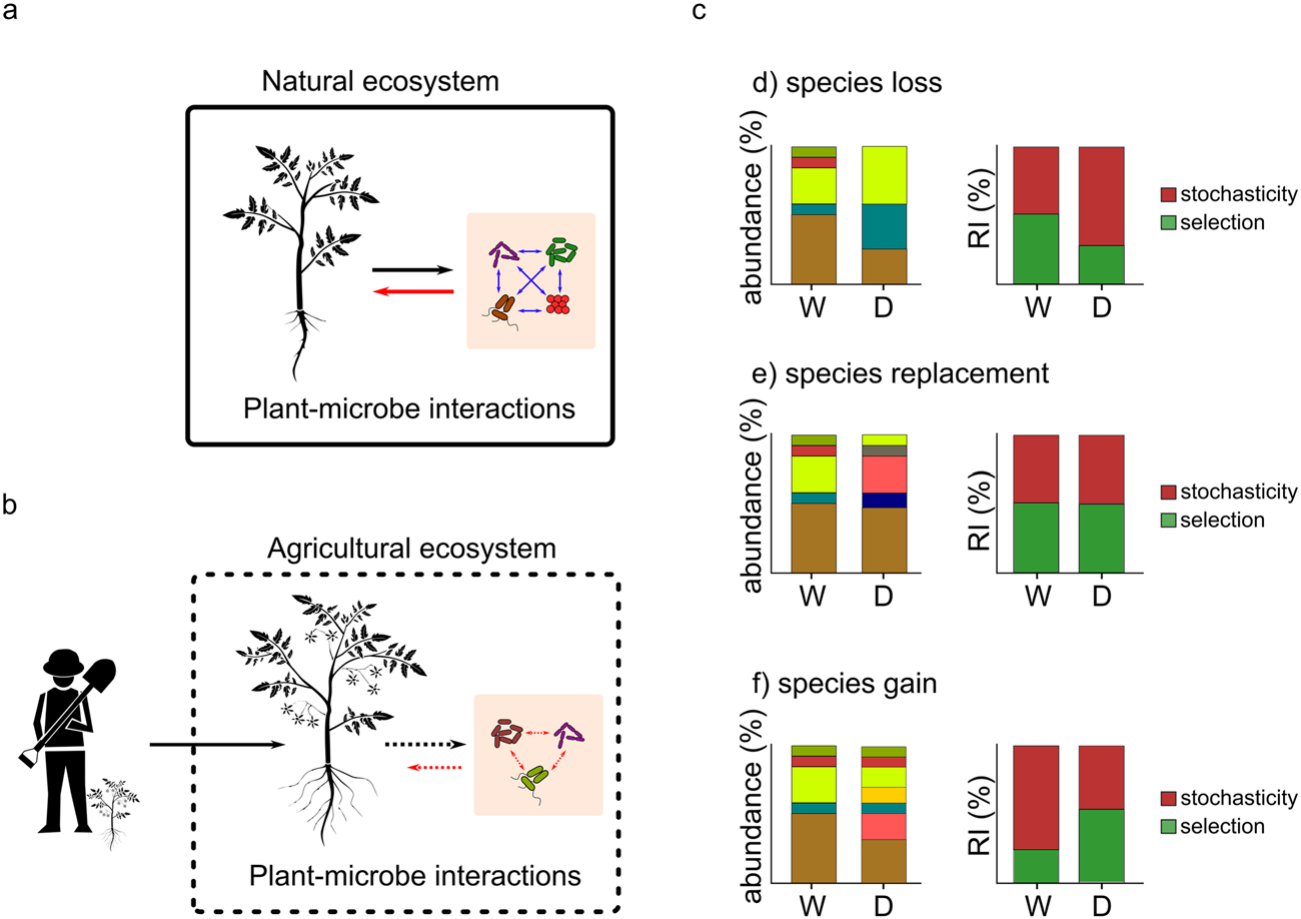
Differences in plant microbiomes between wild and domesticated plants are governed by host-to-microbe and microbe-to-microbe effects dictated by plant domestication. **a** Wild plants are under natural selection to control their microbiota for positive microbe-to-host effects. Black arrow indicates host-to-microbe effects (the leash) and red arrow microbe-to-host effects. Microbe-to-microbe effects are indicated by blue arrows. The black line surrounding host and microbiome represents the natural environment. **b** Domesticated plants are under artificial and indirect selection. The domesticator strongly influences the plant genotype through artificial selection (first leash, black arrow) and indirect selection exerted by the agricultural ecosystem (black dotted line surrounding host and microbiome), so that the positive microbe-to-host effects arising from host control of the microbiome (second leash, black dotted arrow) are reduced (red dotted arrow). Under these circumstances, domesticated plant microbiomes are ecosystems on a double leash from domesticator to host and host to microbe. Additionally, the agricultural ecosystem can affect microbe-to-microbe and host-to-microbe interactions, with consequences for microbe-to-host effects. **cd-f** The double-leash hypothesis could predict species loss (**cd**), replacement (**ce**), and gain (**cf**) caused by different host-to-microbe effects induced by domesticated plant phenotypic traits. Different colours in the relative abundance charts indicate different microbial members. W wild-type plants. D domesticated plants. RI Relative importance of the ecological processes driving community assembly. Plant drawings were downloaded from iStock under standard license agreement.

In a natural environment, plants would be under selective pressure to maximise positive microbe-to-host effects, through control of the host microbiome (the leash)^29^ (Fig. 1a). However, under domestication, host evolution would be also driven by artificial selection and natural (indirect) selection acting through the agricultural ecosystem^31^. Under these circumstances, the host-associated microbiome would become “an ecosystem on a leash” held by the host but modified by the domesticator, who influences the evolution of the host through a second leash (Fig. 1b). The second leash, which is exerted by the domesticator through direct selection and/or indirect selection, mainly acts on the host at a genotypic level. Artificial selection would act by increasing the contribution of the genotypic component in the three-way interaction between genotype, environment, and host microbiome that determines the plant phenotype, to maximise the heritability of artificially selected traits in different environments. Moreover, plant traits directly selected by the domesticator are not necessarily linked to higher fitness, thus we predict a lower contribution of the microbiome to the manifestation of these phenotypes compared to wild progenitors.

Indirect selection would also act at a genotypic level, but traits under indirect selection would confer a fitness advantage to individuals growing in agricultural ecosystems. For this reason, we cannot exclude a contribution of microbe-to-host effects in the manifestation of certain plant traits under indirect selection. Nonetheless, the domesticator could still guide indirect selection through changes in agricultural practices, ultimately limiting the selection of unwanted phenotypes^32^.

Since phenotypic changes caused by artificial selection did not arise as a plant response to control its microbiota for positive microbe-to-host effects, it is logical to predict that changes in the domesticated plant microbiome assembly relative to wild progenitors would be driven by the effect of these artificially selected phenotypic changes, which may not necessarily favour host control of the microbiota to the benefit of the host, with a few notable exceptions such as artificial selection for disease resistance. This does not suggest weaker host-to-microbe effects (Fig. 1b), but simply an effect mainly dictated by plant phenotypes directly selected through artificial selection on the microbiome. Therefore, while the plant will still exert control of its microbiota, artificial selection would lower the effect of the plant leash, and host-microbiome assembly will be shaped by the genotypic leash exerted by the domesticator.

When phenotypic changes are induced by indirect selection, host control of the microbiota could still favour positive microbe-to-host effects. Therefore, indirect selection would not necessarily lower the effect of the plant leash, but potentially drive a different microbiome assembly compared to one of the wild progenitor that confers positive microbe-to-host effects. However, several studies have indicated a reduction in positive microbe-to-host effects in domesticated hosts compared to wild progenitors, including studies involving wheat, maize^33,34^, and and soybean^35^ (reviewed in ref. ^36^), possibly suggesting a weak effect of indirectly selected plant traits in determining microbiome assembly.

The results of host-to-microbe effects dictated by domesticated plant phenotypes could lead to microbial species loss, gain, and species replacement depending on the ecological processes driving host-microbiome assembly (Fig. 1c). When host-to-microbe effects caused by domesticated plant phenotypes lead to species loss, and the lost microbial species were selected through host-to-microbe effects in wild progenitors, then a decrease in the overall importance of selection in driving community assembly compared to wild plants is expected (Fig. 1cd). Our double-leash framework predicts this could happen when domesticated plant phenotypes are the result of artificial selection. Species loss could also be caused by host-to-microbe effects induced by plant traits under indirect selection, where the lost microbial species negatively affect plant fitness under the agricultural ecosystem. Moreover, the agricultural ecosystem could also influence microbe-to-microbe effects, potentially influencing the outcome of host-to-microbe effects.

When domesticated plant traits exert strong host-to-microbe effects, species replacement and/or species gain could dominate the difference between wild and domesticated plant microbiomes. In the case of species replacement, there could be no difference in overall selection exerted by the wild and domesticated plants (Fig. 1ce). However, the double-leash hypothesis would predict that wild and domesticated plants would be associated with different microbial communities that exert different microbe-to-host effects. In the case of species gain, an increase in the overall importance of selection in driving community assembly compared to wild plants is expected (Fig. 1cf). Both species replacement and species gain are predicted to be an effect of domesticated plant phenotypes under artificial or indirect selection. However, we might expect that under indirect selection, the resulting microbiome is more likely to provide positive host-to-microbe effects.

To date, research studies seem to agree with the predictions of this framework, particularly in relation to species loss and species replacement and reduced selection caused by plant domestication. For example, this has been shown for the wheat rhizosphere microbiome as a consequence of plant dwarfing^37^ and also for the wheat seedling microbiome^38^.

## The effect of agricultural practices

As host control of the microbiome in domesticated plants acts in the context of agricultural ecosystems, we need to also contextualise their effect on microbe-to-microbe interactions during domestication, which is a multi-staged process in which both host genotypes and environments have changed^31^. During the early stages of domestication, plants and their microbiome were adapted to their geographically local, predictably unstable environment, not yet fully agricultural. The differences, if any, in the plant microbiomes of wild and early domesticated crops would be the result of variation in interactions between host, environment, and microorganisms mainly caused by host genetic and environmental differences. Differences in host control of the microbiome in domesticated plants compared to wild plants would arise through artificial and indirect selection but agricultural practices would exert a low influence on microbe-to-microbe effects.

During later stages of the domestication process plants were grown over a wider geographical area and plant genotypes adapted to different agricultural environments (landraces), and new cultivars were bred. During these stages, host-to-microbe effects would be influenced by the double leash but also by differences in environment-to-host and microbe-to-microbe effects caused by different agricultural ecosystems and new environments. For example, the effect of agricultural practices, particularly intensive fertilisation and pest control methods could act to lower soil microbial diversity^39,40^, influencing the outcome of host-to-microbe effects.

Below, we further discuss these effects for the plant compartment that has shown the most remarkable phenotypic changes, the seed.

## The seed provides an ideal context in which to study the effect of plant domestication on plant microbiomes

The seed microbiome and plant–microbe interactions in the spermosphere have important ecological functions (for recent reviews see refs. ^41,42^). Seed-associated microorganisms can be inherited through vertical transmission^43–48^; they provide beneficial effects for seedling growth and establishment^23,43,49–52^ and determine, together with soil, the rhizosphere microbiome^53^. Moreover, artificially introducing plant growth-promoting bacteria into seeds has been shown to increase wheat crop yield^54^, opening the possibility of a new era of bacterial inoculants and plant breeding^55^.

Plant domestication has influenced and changed many seed-related phenotypes, and while root architecture has also changed from wild to domesticated crops^56^, the increase in seed size exceeds any other difference noticeable in roots. For example, maize (*Zea mays* ssp. *mays*) kernel weight has increased more than 10 fold compared to teosinte (*Z. mays* ssp. *parviglumis*)^57^. If the same effect was to be observed in roots, domesticated maize would have on average 8 m long roots. Differences in root length have already been linked to differences in the composition of the rhizosphere bacterial community between wild and domesticated common bean^58^ but the remarkable increase in seed size of domesticated plants and its consequences for plant–microbe interactions has thus far been neglected. Seed chemical composition has also changed through plant domestication^59^, with unknown consequences for the seed microbiome and plant–microbe interactions in the spermosphere (the area surrounding a germinating seed that is influenced by the seed).

We propose that under the double-leash framework, seeds represent an ideal model to study the effect of domestication on plant microbiomes. Seeds are often the main target of direct and indirect selection and they often showcase the most remarkable phenotypic changes associated with domestication compared to other plant compartments, such as roots. Seed microbiomes are characterised by low alpha diversity values, potentially favouring host control of microbial communities^29^. Therefore, the strength of the double leash in shaping microbial communities could be stronger for the seed compartment.

Seeds are also involved in the vertical transmission of microbes. For example, this has been widely reported for the fungal symbiont *Neotyphodium* in grasses^45,60^ and also for members of the bacterial microbiota in maize^46^. Vertical transmission, or inheritance, is important because it can favour a higher degree of co-evolution between host and symbionts^61^ (although see ref. ^62^). Vertically transmitted microorganisms could increase the fitness of their host by promoting competition and seed establishment, thus increasing the chances of seedlings developing into mature plants^23,49,63^. The effect of phenotypic changes (host-to-microbe effects) caused by plant domestication on vertical transmission remains unknown.

### The effect of domestication on seed microbiome assembly: the double-leash paradigm

**Fig. 2:**
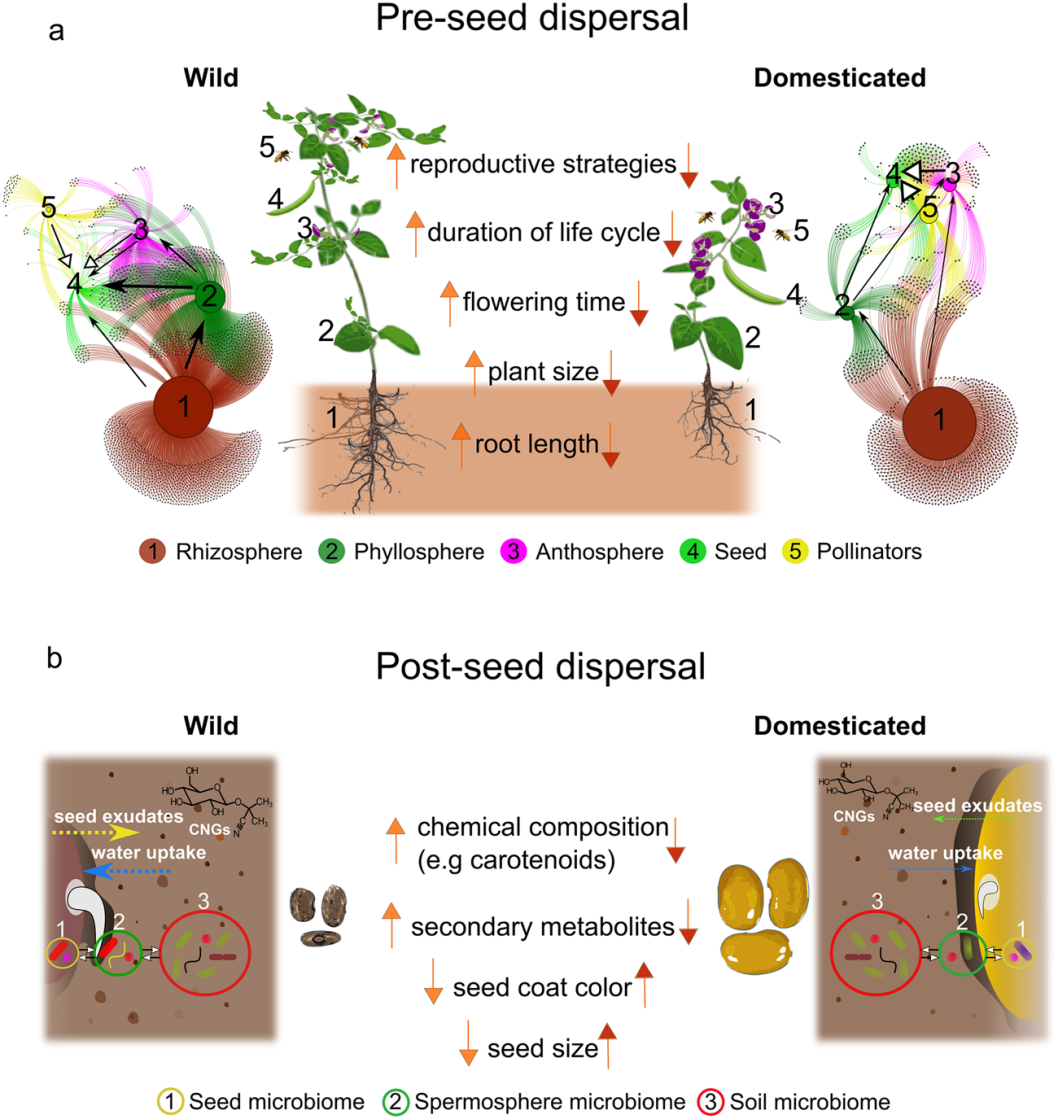
Phenotypic changes introduced with plant domestication have consequences for the microbial connectivity between different plant compartments and the spatial and temporal dynamics of plant–microbe interactions in the spermosphere. **a** Pre-seed dispersal: Phenotypic changes caused by plant domestication affect both the below and above-ground plant compartments. In wild plants, we propose that the microbial connectivity (sharing of microbial members) between the environment and plant compartments, visualised as a bipartite microbial network, could reflect a higher degree of vertical transmission of microorganisms to plant seeds (represented by wider black arrows), under the influence of host-microbiome control mechanisms. Vertical transmission can occur from the rhizosphere (1) to the seed compartment (4); from the rhizosphere (1) and phyllosphere (2) to the seed compartment (4); or from the rhizosphere (1), phyllosphere (2) and anthosphere (3) to the seed compartment (4). Horizontal transmission (white arrow) can occur from plant to plant in the rhizosphere and phyllosphere via air-borne, soil-borne and water-borne inocula, vectors, and mechanical transmission (not illustrated), from pollinators (5) to the seed compartment (4) or from the anthosphere (3) to the seed compartment (4). We speculate that domesticated plants exhibit differences in microbial connectivity compared to their wild counterparts. In this example, we predict higher importance of horizontal transmission (represented by wider white arrows) in the seed microbiome assembly. **b** Post-seed dispersal: Phenotypic changes caused by plant domestication can affect the spatial and temporal dynamics of plant–microbe interactions in the spermosphere. Wild plants have smaller seed sizes and different colours of the seed testa compared to domesticated plants. Water uptake may be negatively correlated with seed or seed pore size and seed colour is linked to morphological features of the seed testa influencing imbibition rate. The dynamics of seed germination, imbibition and seed exudation differ from domesticated plants. Secondary metabolites such as cyanogenic glycosides (CNGs) and antimicrobial proteins may also be more abundant or present at higher concentrations in seeds of wild plants. We speculate that these phenotypic changes are likely to lead to different spatial and temporal dynamics of interactions between the seed microbiome (1), the spermosphere microbiome (2), and the soil microbiome (3). In the example shown, seeds of domesticated plants germinate slowly, resulting in a different spermosphere microbial community compared to wild plant seeds. This situation could be reversed for seeds of wild plants that manifest dormancy. In this case, seeds of domesticated plants would germinate faster, regardless of seed size.

In our proposed view, domesticated plant microbiomes are ecosystems on a double leash, which can be exemplified by consideration of the seed microbiome. Changes in the seed-microbe interactions of domesticated plants compared to wild progenitors could arise through host-to-microbe effects acting pre-dispersal (when the seed is developing), such as changes in the plant compartments and routes used by microorganisms to colonise seeds (Fig. 2a), as well as through the biochemical and physical properties of the developing and mature seed, which act both pre-seed and post-seed dispersal (Fig. 2b). We discuss the mechanisms and potential impact of both of these interactions in more detail below.

### Host-to-microbe effects of plant domestication on seed microbiome assembly (pre-seed dispersal)

Under our double-leash framework, the domesticator has influenced and changed plant traits that have led to host-to-microbe effects that differ from those occurring in the wild plant progenitors. The phenotypic changes caused by domestication and their effect on seed microbiome assembly could be represented as changes in microbial connectivity (sharing of microbial members), between plant compartments and the environment (Fig. 2a). Even though the exact routes used by microorganisms to colonise seeds remain under-investigated, it is now widely recognised that the seed microbiome can originate from the anthosphere, rhizosphere, and phyllosphere, each of which could exert host–microbe effects on the composition of the seed microbiome (for recent reviews see refs. ^42–44,64^).

The colonisation of seeds via the anthosphere is particularly well documented for plant pathogens. For example, *Xanthomonas euvescicatoria* infects pepper seeds through the flower stigma^65^. Similarly, *Acidovorax citrulli* can colonise seeds of watermelon through a floral pathway^66^. Pollen can also be a source of microorganisms, potentially transmitted to the seeds through the flower stigma and style. For example, *Pseudomonas syringae* pv. *actinidiae* colonises the anthers and pollen of infected male plants^67^. In turn, the microbiome of flowers and pollen is influenced by the visits of pollinators^68–71^, which could also be a source of potentially horizontally transmitted microbes to the seeds. For example, *Lactobacillus*, *Spiroplasma*, and *Frischella* genera found on honey bees were found to be more abundant in the seed microbiome of bee-pollinated compared to hand-pollinated oilseed rape^72^. Multiple phenotypic changes observed in domesticated plants could have direct and indirect consequences for seed colonisation via the anthosphere, including earlier flowering time, flower size, colour and shape, and floral reward chemistry^73^. Changes in floral chemical composition and flowering time are known to lead to different flower-pollinator interactions, and potentially to differences in the importance of horizontal transmission in contributing to the seed microbiome^72^.

Transmission of bacteria via the vascular system from the rhizosphere and other plant tissues is also known to be important for seed colonisation. For instance, co-localisation of bacteria with plant vascular tissues of seeds has been demonstrated for seeds of *Anadenanthera colubrina*^74^ and bacteria potentially originating from soil were found to be endophytic on flowers, berries, and seeds of grapevine^75,76^. Rodriguez et al.^77^ suggested that the soil is likely to be an important reservoir of microorganisms that contributes to the seed microbiome assembly of *Setaria viridis*. The authors conducted an experiment growing *Setaria viridis* in a sterile substrate for successive plant generations and detected an altered and simplified seed microbial community, concluding that at least part of the seed microbiome could originate from the soil. Similar findings were observed for the seed microbiome of *Prosopis velutina*^78^. The shorter life cycles typical of domesticated plants could reduce connectivity between the rhizosphere and seed microbiomes, lowering microbial inheritance; also changes in root exudates, root architecture and plant immunity are likely to substantially influence the ability of rhizosphere and phyllosphere-colonising organisms to colonise seeds.

As host-to-microbe effects caused by domestication have directly or indirectly influenced the colonisation routes used by microorganisms to colonise seeds, thereby affecting the microbial connectivity between different plant compartments and the environment, the seed microbiome of wild progenitors is likely to harbor different members of the microbiota or to show differences in community composition when compared to that of domesticated plants^27^. However, the extent to which different flowering times, duration of plant cycles, and even changes in reproductive strategy affect the seed microbiome assembly remains to be elucidated. It is also important to bear in mind that the ability of microorganisms to reach the seed via the vascular system, or other routes, is not sufficient for these organisms to become established in the seed, as antimicrobial proteins and secondary metabolites present in the developing seed limit microbial colonisation. Therefore, another unknown effect acting on the seed microbiome both pre-and post-dispersal is whether the reduction in secondary metabolites often observed in seeds of domesticated crops^79,80^, along with other changes in seed chemistry influences seed microbiome composition and viability. As many plant pathogenic microorganisms are transmitted via seed, it will be particularly important in future studies to investigate whether changes in seed properties associated with domestication positively or negatively affect the ability of specific microorganisms to survive the dispersal stage and be transmitted to the emerging seedling, or even to transition along the free-living/parasite/mutualist continuum^81^.

Another aspect of plant domestication which may influence the final pre-dispersal assembly of the seed microbiome is grafting. Grafting has been used for thousands of years to foster the domestication of fruit trees^82^. Many domesticated plants are grown as grafted plants^82^, such as several horticultural annual species and fruit tree species, including grapevine. It has been shown that rootstock type influences the rhizosphere and root endosphere microbiome in grapevine^83^ and tomatoes^84^, possibly leading to changes in microbial connectivity between plant compartments. Grafting can occur in nature, but it is used as a systematic agronomic practice in many domesticated species, thus potentially contributing as a factor steering the seed microbiome assembly by changing the microbial connectivity between plant compartments.

### Host-to-microbe effects of plant domestication on plant–microbe interaction in the spermosphere (post-seed dispersal)

As mentioned above, modern crops usually have larger seeds compared to their wild ancestors, even in crops in which seeds are not directly consumed by humans or livestock, accompanied by changes in chemical composition. To what extent this change may have affected the seed-associated microbiome and plant–microbe interactions in the spermosphere, remains unknown. It is not within the scope of this article to discuss why domestication has led to plants with bigger seed mass, but this is thought to be the result of both indirect and artificial selection, and is observed in both grain crops and in crops that are not harvested for their grains. Indeed, a seed mass increase has been reported for lettuce, carrots, and potatoes, and is thought to have been partially driven by unconscious selection in which indirect selection was exerted by agricultural practices^10^.

As a result, domestication has altered resource allocation and the trade-off relationship between seed mass (SM) and seed output (SO)^85,86^. If a fixed amount of energy has to be devoted towards the production of seeds, then a plant can either produce a large number of small seeds, or a small number of larger seeds^87^. Overall, domesticated crops tend to have larger SM and smaller SO compared to wild plants^86^ (for further reading on the ecological implications of seed size see refs. ^88–90^). We speculate that this change in seed size is likely to play an important role in determining the spatial-temporal dynamics of plant–microbe interactions in the spermosphere. The concept of the spermosphere was fully developed by Onorato Verona in 1958^91^ and since then, numerous studies have investigated the importance of this dynamic plant compartment (for comprehensive reviews see refs. ^41,92^). Eric Nelson, in his review^92^ wrote: “*The carbon released from seeds during germination represents the major driving force behind plant-microbe and microbe-microbe interactions in the spermosphere*”. Therefore, seed size, which may be directly associated with differences in the amount and/or the nature of carbon released, may directly affect the microbial interactions of the spermosphere. Additionally, if seed enlargement, driven by domestication, alters the dynamics of water uptake, in turn, this could influence the deposition of seed exudates. In soybean, small seeds imbibe and germinate faster compared to larger seeds, regardless of the moisture levels^93^. Similar negative correlation trends between seed size, water uptake, and germination time were also observed for maize^94,95^ and potatoes^96^.

A more detailed study investigated why smaller seeds have a higher percentage of water uptake in soybeans^97^. The authors reported that in soybean smaller seeds have large rounded pores whereas medium and large seeds have smaller and elongated pores. Pores in small seeds were open and functional, whereas they were distorted and plugged in large seeds. As microbial interactions in the spermosphere are influenced by seed exudates^92^, the release of which depends on imbibition^98,99^, we speculate that seed and pore size could affect the dynamics of these interactions.

We are still far from understanding what compounds, present in seed exudates, are used by microorganisms to proliferate in the spermosphere. Nonetheless, we know that the spores of *Pythium ultimum*, a seed-infecting pathogenic oomycete, are stimulated to germinate rapidly in the presence of oleic and linoleic acids, which are fatty acids commonly found in seed exudates^41^. Also, the quantitative aspects of seed exudation are important. For example, *Enterobacter cloacae*, a spermosphere-colonising bacterium, has higher metabolic activity on pea seed exudates than on cucumber seed exudates. The latter has an order of magnitude fewer nutrients^100^. Barret^101^ reported that germination influences the structure of the microbiota and that the observed shift in composition between seed microbiota and seedling microbiota was associated with a higher relative abundance of bacterial (*Bacillus, Massilia, Pantoea*, and *Pseudomonas*) and fungal (*Trichodermam*, *Chaetomium*) taxa that exhibit fast growth rates.

Together with seed size, seed colour, which is linked to morphological features, has been linked to the dynamics of seed imbibition. For example, in proso millet (*Panicum miliaceum*), darker seeds have heavier seed coats, imbibe and germinate more slowly and suffer less imbibition damage^102^. In *Phaseolus vulgaris*, cultivars with white seed coats imbibe faster and have higher solute leakage compare with seeds with a darker colour^103^. As domestication has not only caused seed enlargement but also expanded the colour range of seeds^5^, and both seed size and colour affect imbibition and germination, they should be considered together when assessing whether there is an effect on the spermosphere microbiome. We propose that one of the consequences of the production of large seeds, which may have slower imbibition and germination rates compared to smaller seeds, is different temporal dynamics of spermosphere–microbe interactions. The consequences of these different temporal dynamics of spermosphere colonisation for seedling establishment and plant growth remains unexplored.

Similar to seed size and seed colour, seed chemical composition has also been affected by domestication. For example, domesticated lima bean (*Phaseolus lunatus*) seeds have been reported to have up to 20 times lower concentrations of cyanogenic glycosides than seeds of wild populations^80^, and legume seeds of domesticated plants have fewer carotenoids than their wild counterparts^104^. Moreover, mineral composition can also differ between wild and domesticated seeds. For example, calcium concentration was found to be negatively correlated with seed mass in common bean seeds^105^ and minerals have been reported to influence microbiome assembly in the rhizosphere^106^.

## Testing the double-leash framework

In general, our double-leash hypothesis predicts that domesticated plant traits, either under direct or indirect selection, explain the difference in ecological processes driving community assembly between wild and domesticated plants. The effects of domesticated plant traits resulting from direct and indirect selection on host-microbiome assembly could lead to species loss, species gain, or species replacement (Fig. 1c). To test the double-leash hypothesis, researchers could perform experiments in which wild and domesticated plants are grown in a greenhouse with non-native soil (to avoid an effect of plant adaptation to agricultural or natural ecosystems). The selection of wild and domesticated plants should be performed to provide a wide range of phenotypic traits or researchers could create experimental populations (e.g. from a cross between a domesticated and wild accession of the same species) that are segregating for a certain domestication trait. For example, to study the effect of seed size on seed microbiome assembly, accessions of both wild and domesticated plants should be selected that showcase a wide range of seed sizes.

The double leash could then be tested by looking at significant correlations between the plant phenotype distance matrix and the distance matrix calculated from the relative importance of ecological processes driving microbiome assembly. This approach has been already used to understand the environmental factors driving microbiome assembly in grasslands as a response to warming^107^. If the plant trait(s) under selection during domestication (e.g. flowering time, root length, seed size) is significantly correlated with the observed relative importance of selection, then the double-leash hypothesis is supported.

This approach, in which ecological processes driving microbial community assembly are broken down into their components, namely stochasticity and selection, and explained by domesticated plant phenotypes, provides a quantitative approach to identify the causes of differences in microbiome assembly and thereby test the double-leash hypothesis. However, it should be noted that this approach rests on the assumption that host selection acts on microbial community assembly. The double-leash hypothesis would not predict the outcome of assembly processes mainly governed by stochasticity (weak selection), for example when hosts exert low selection on microbial communities (weak host-to-microbe effects).

## Biotechnological implications of the double-leash framework

Understanding how to re-introduce lost microbial species that exert positive microbe-to host effects in domesticated plants is an important and current area of research^36,108^. Porter et al.^36^ suggest the possibility to re-introduce into domesticated plant genomes loci responsible for symbiosis, similarly to the introgression of genes responsible for disease resistance^109^, which have been lost during domestication. Similarly, other studies suggest acting at a plant genotypic level to re-establish positive plant–microbe interactions^110^. Our framework, focusing on the effect of plant phenotypes on driving microbiome assembly, does not focus on microbe-to-host effects but tries to understand whether a certain plant phenotype affects the selection of microbial species. This suggests an alternative approach for enhancing plant productivity, based on the identification of microbial groups that are under positive selection in the context of the double leash, which can be screened or engineered for positive host-to-microbe effects. While this approach presents several limitations at the moment, including challenges associated with the release of GMOs in the environment, advances in bacterial genome editing and biocontainment make this an increasingly feasible approach for developing synthetic plant growth-promoting communities.

## Conclusion and future directions

Domesticated plants may have lost, or show a reduction in the ability to establish beneficial associations with microbial communities through changes in host–microbe or microbe–microbe interactions^33,34^. However, how host control of the microbiota is influenced by domestication through different host-to-microbe effects remains poorly understood. We advocate that by extending the concept of the host microbiome as an ecosystem on a leash to a double-leash framework that includes the domesticator and the consequences of direct and indirect selection, host-to-microbe effects can be framed in a more comprehensive perspective for understanding their role in determining the assembly of domesticated plant microbiomes. Moreover, our double-leash hypothesis could lead to the identification of microbial species selected by host-to-microbe effects driven by domesticated plant phenotypes that could be applied or engineered for positive microbe-to-host effects. To that end, the seed compartment is not only emblematic in terms of the phenotypic changes caused by domestication but also an ideal candidate to investigate the consequences and potential for exploitation of host-to-microbe effects. In the long term, unveiling the mechanism and consequences of domestication of the plant microbiome, and in particular the seed microbiome, can help to explain new adaptative patterns of plants and provide new insights toward more sustainable management of arable land.
